# A long-term dataset of topography and nearshore bathymetry at the macrotidal pocket beach of Porsmilin, France

**DOI:** 10.1038/s41597-022-01170-3

**Published:** 2022-03-11

**Authors:** Stéphane Bertin, France Floc’h, Nicolas Le Dantec, Marion Jaud, Romain Cancouët, Marcaurélio Franzetti, Véronique Cuq, Christophe Prunier, Jérôme Ammann, Emmanuel Augereau, Stevenn Lamarche, Déborah Belleney, Mathias Rouan, Laurence David, Anne Deschamps, Christophe Delacourt, Serge Suanez

**Affiliations:** 1 UMR 6538 Laboratoire Géosciences Océan, Univ. Brest-CNRS, Institut Universitaire Européen de la Mer, Rue Dumont d’Urville, 29280 Plouzané, France; 2 UMS 3113 Institut Universitaire Européen de la Mer (IUEM), Univ. Brest-CNRS, Rue Dumont d’Urville, 29280 Plouzané, France; 3 UMR 6554 Littoral, Environnement, Géomatique, Télédétection, Univ. Brest-CNRS, Institut Universitaire Européen de la Mer, Rue Dumont d’Urville, 29280 Plouzané, France

**Keywords:** Physical oceanography, Geomorphology, Geophysics, Geodynamics, Sedimentology

## Abstract

Long-term datasets documenting the evolution of coastal forms and processes, through the provision of recurring beach as well as shoreface morphological observations and accompanying time-series of environmental controls, remain difficult to collect and are rarely made available. However, they are increasingly needed to further our understanding of coastal change and to improve the models that will help planning what our future coast will be. This data descriptor presents the results of topographic and bathymetric surveys at Porsmilin, a macrotidal embayed beach situated in Brittany, northwest France. The Porsmilin beach survey program was launched in January 2003 by the Institut Universitaire Européen de la Mer (IUEM/Univ. Brest) and is continuing today in the framework of the French coastal observation service SNO-DYNALIT. The dataset contains over 16 years of monthly beach profile surveys and a large collection of repeated high-resolution subtidal and subaerial digital elevation models (DEMs). The dataset is accompanied by time-series of inshore waves and water levels, and enriched metadata, that will facilitate its future reuse in coastal research.

## Abstract (summary)

**Table:** 

Measurement(s)	Beach topography • Nearshore bathymetry
Technology Type(s)	RTK-GNSS • Terrestrial laser scanning • Digital photogrammetry • Multibeam echosounder
Factor Type(s)	Elevation
Sample Characteristic - Environment	shore
Sample Characteristic - Location	Bretagne Region

## Background & Summary

Monitoring coastal morphodynamics at representative sites over time scales that span several years or even decades is necessary to further our understanding of natural^1–5^ and human causes^6–9^ of coastal change, to develop beach evolution models^10–14^ that will prove reliable with regards to observations, and hence to help adapting coastal planning strategies to future changes^15–18^. Achieving all these objectives is generally impeded as long-term datasets documenting the evolution of coastal forms and processes, for instance through providing repeated beach and shoreface morphological observations and accompanying time-series of environmental forcing conditions, remain difficult to collect and are rarely made available.

Recently, coastal monitoring programs at a limited number of sites worldwide have started opening their collections, allowing free and unrestricted access to the data and facilitating their reuse through data descriptors^19–22^. These datasets are essentially the results of topographic surveys, eventually accompanied by a few subaqueous profile surveys. Other studies report on the use of long-term subaqueous profile surveys obtained along the coasts of Japan, the Netherlands and the USA^23–25^.

Although methods for measuring coastal bathymetry changes have significantly improved^26,27^, previous subaqueous surveys are essentially limited to single or spaced profiles. Besides, when attempted for wave-dominated coastlines, these subaqueous surveys may not achieve sufficient depth to include the seaward limit at which morphological change becomes non-significant over a typical year (i.e., the closure depth^28^). As a result, despite the examples above, there is a paucity of data that provide both the high spatial and temporal resolution necessary for capturing subaerial beach morphology and dynamics, and that are capable at the same time of quantifying sediment exchanges with the subtidal zone.

Pockets and embayed beaches are geologically constrained morphological cells that are common along rocky coasts and as a result of beach compartmentalisation with groynes. They are characterised by indented geometries imposed by geological constraints or structures, which, in interaction with waves, can result in beach rotation^29,30^ and bolster the formation of rips and alongshore-variable 3D morphologies^31^. Observations of the predominantly cross-shore forcing of the beach sediment have suggested that these beaches function as semi-enclosed sediment compartments most of the time, with sediment bypasses occurring in the subtidal zone during major storms^28,32^. Monitoring programs of pocket and embayed beaches have consisted mainly in video-derived shoreline positions and beach profiling^30,31^, which can produce high-frequency data over long periods of time (i.e., several years or decades), but provide limited quantitative hindsight on sediment transfers at the full embayment scale.

**Fig. 1:**
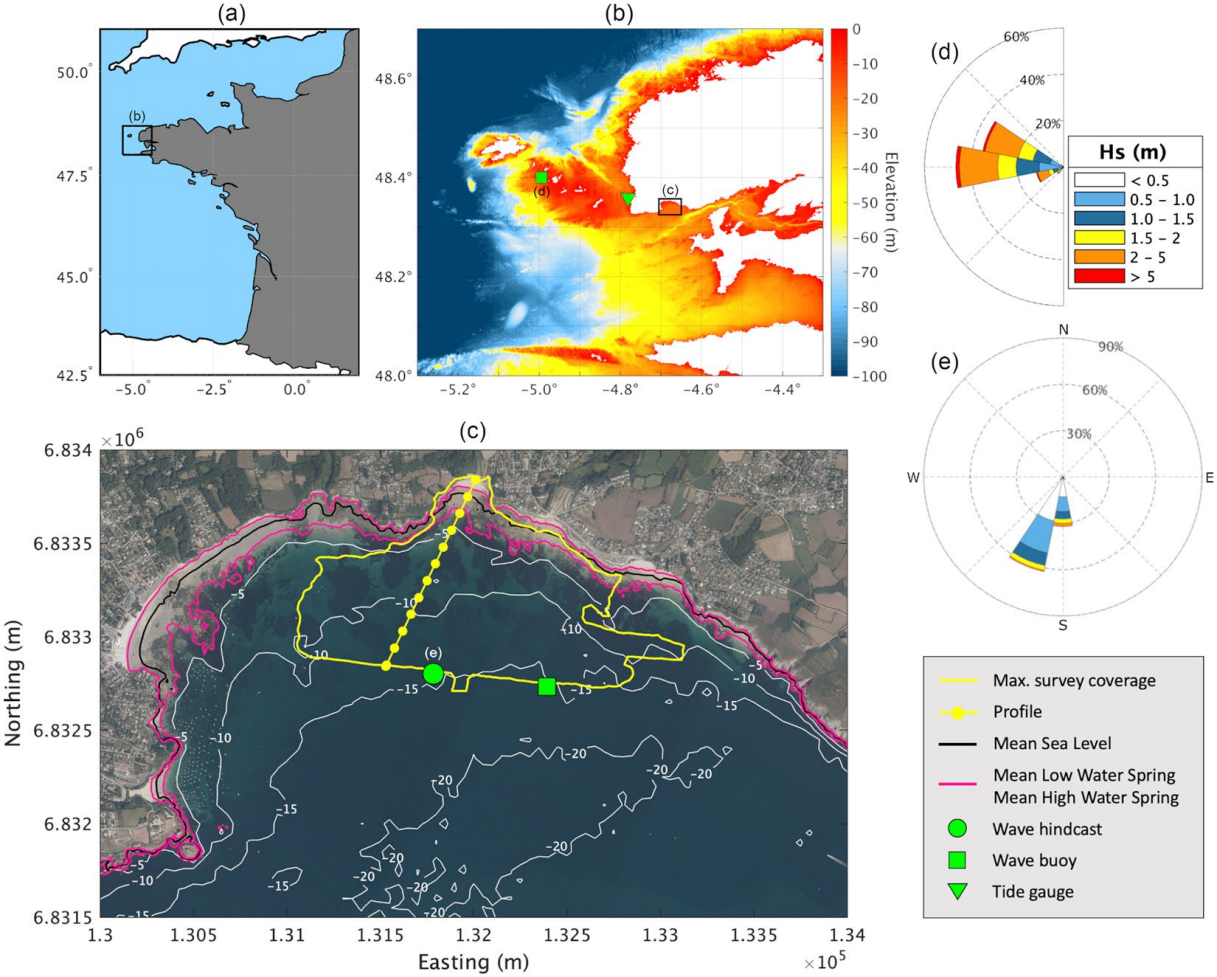
Survey site. (**a**) Map of western France. (**b**) DEM of northwest Brittany coastline (source: MNT Bathymétrique de façade Atlantique^67^ (Projet Homonim) - Shom) showing the location of Pierres Noires CANDHIS wave buoy (node 47039) and Le Conquet tidal gauge (green square and triangle, respectively). (**c**) Orthophotograph of Bertheaume Bay and Porsmilin beach (source: Ortho Littorale V2 - Ministère en charge de l’environnement), showing the maximum survey coverage and reference profile line with graduations every 100 m (yellow), depth contours at 5 m intervals (grey, source: Litto3D Finistère 2014 - Shom) and contour lines corresponding to mean sea (black) and spring tidal levels (pink). Green circle and square represent the virtual buoy (node 47554) and the Porsmilin CANDHIS wave buoy (node 47562), respectively. Coordinates are relative to the French metropolitan coordinate system Lambert 93. (**d,e**) Wave roses for Brittany deepwater wave climate and Porsmilin, respectively. Deepwater wave data are based on waves measured bi-hourly at Pierres Noires between 2005 and 2019. Inshore wave data are based on hourly hindcasted waves (node 47554) between 1994 and 2019.

In this paper, we describe for the first time the long-term dataset of beach topography and nearshore bathymetry at Porsmilin, a macrotidal embayed beach situated in Brittany, northwest France (Fig. 1). The dataset represents one of the longest records of continual beach surveys along Europe’s Atlantic seaboard^33,34^. The Porsmilin beach survey program was launched by the Institut Universitaire Européen de la Mer (IUEM, Univ. Brest) in January 2003, and initially consisted in frequent beach profile surveys. From 2008, it evolved to also incorporate recurring high-resolution (0.5 m) subaerial and subtidal DEMs obtained using a combination of modern remote sensing techniques, and whose spatial coverages encompass the regions of significant bed changes.

Field data collected at Porsmilin have been used to investigate the morphodynamic response of macrotidal embayed beaches^31,35^, equilibrium modelling of the beach profile^36^, and the conditions controlling beach cusps development^37^. They also contributed to a regional assessment of the shoreline dynamics of the Brittany coast^38^, and erosion and recovery following extreme storm activity during the 2013–2014 winter at both local^39^ and European scales^33,34^. Other studies interested in the methods for collecting and processing these geomorphic data present detailed information and validation of the techniques implemented in the survey program^40–42^.

Starting in 2019, after reaching sixteen years of running the Porsmilin survey program, the dataset was completely revamped with the aim that unrestricted access to the complete and archived data would follow. Doing so allowed for the incorporation of recent advances in geomorphic processing and validation techniques, providing consistent and well-documented data that will be easily reused by others for their analyses. Accompanying the results of our field surveys, we provide hourly wave hindcasts and tidal levels, obtained with the help of external organisations, for the period 2000–2019.

## Methods

### Site description

The approximately 2470 km-long Brittany coastline in northwest France protrudes into the Atlantic Ocean and abuts the English Channel to the North (Fig. 1a,b). This jagged coastline chosen by Mandelbrot to illustrate fractals in nature^43^ is essentially rocky and contains beaches of moderate size (42% are shorter than 200 m and only 12% are longer than one km, based on a sample of 600 beaches^38,44^), often characterised by moderately to highly indented planform geometries.

Situated at the entrance of the Bay of Brest, Porsmilin is a narrow (200 m long), moderately indented, macrotidal embayed beach nested in the wider Bertheaume embayment and facing south-southwest (Fig. 1). The sandy beach is flanked by cliffs on both sides (~15 m tall, orthogneiss and diabase composition) and is backed by small dunes (~1-2 m tall) separating the beach from a brackish water marsh. The dunes have been repeatedly reshaped by storms and increasing human interventions since at least the 1940s. The intertidal beach is also bounded East and West by bedrock reefs, uncovered at low tide and extending offshore, and by a small headland to the West. Eastward, part of the shoreline is backed by a rip-rap protected seawall remnant of WW2 abutting a carpark. Other human alterations include a small boat ramp and a water outlet pipe eastward of the beach, where a small stream was previously running. The semi-enclosed configuration, together with a shoreline orientation roughly parallel to incoming waves, accompanies a predominantly cross-shore forcing of the beach sediment^31,35,36^. The beach typically consists of a berm during summer months, alternating with semi-persistent sand bars forming in the intertidal and subtidal zones^31^. The average beach gradient ranges from 0.02–0.04 on the shoreface to 0.04–0.08 on the intertidal and upper beach. Beach cusps regularly form in the swash zone during higher tides, as a result of wave action, and present typical length scales ~20–40 m.

Sediment samples collected across the beachface are characterized by medium-grained quartz sand (D_50_ = 0.32 mm), with some cross-shore variability associated to coarser sediment near the crest of intertidal and swash bars (D_50_ = 0.7 mm)^45^. Cobble patches are exposed intermittently, principally on the upper beach. Likewise, peat outcrops can be seen near the low-tide water line following energetic waves and erosion of the sand. Using such basal peat deposits, dating along the coast of western Brittany shows that the first generation of sand dune formations initiated from ca. 4000 cal BP (calibrated year before present), with the slowing down of relative sea level rise. At Porsmilin, the onset of present-day dunes was dated to around 770 cal B.P., with a last phase of stabilization dated to ca. 350 cal BP^46,47^.

The deepwater wave climate is highly energetic in this part of the Atlantic Ocean called the Iroise Sea, with a mean Hs ~2.0 m and Tp ~10 s estimated in 60 m water depth using the Pierres Noires wave buoy (Fig. 1b,d). Yet, due to a prominent continental shelf with numerous islands and reefs, which affect wave propagation, wave exposure at the coast is very heterogeneous^38^. The wave climate also exhibits strong seasonal change in wave energy. This generally traduces by relatively calm seas during summer (mean Hs ~1.4 m, Tp~9 s) contrasting with powerful winter storms (10.2 m and 12.3 m significant wave heights for 1 and 10-year storm return periods, respectively). Throughout a typical year, 47% of swell waves originate from a W direction, 36% are from the WNW and 17% from the WSW (Fig. 1d). Superimposed on seasonal modulations, longer-term trends in wave climate represented by North Atlantic Oscillation (NAO) and West Europe Pressure Anomaly (WEPA) indices are responsible for large fluctuations in winter wave energy, which can potentially traduce by the occurrence of exceptional winters in terms of storminess and storm clustering^33,48,49^.

Due to its orientation away from dominant storm tracks, waves are significantly reduced when they reach Porsmilin (mean Hs ~0.7 m estimated in 15 m water depth, Fig. 1c,e), which tends towards the lower-energy low-tide terrace beach state^31,36,50^. Tides are macrotidal and semidiurnal with a mean neap and spring tidal range of 2.7 and 5.7 m^51^, respectively. Despite a relatively sheltered location, the beach adjusts quickly to changes in hydrodynamic conditions with morphological proxies observed to change over a tidal cycle during energetic waves^31,37^. Over the duration of the monitoring program, severe erosional events occurred, the most notable being during the 2013–2014 boreal winter, which was among the most energetic since at least 1948 for most of the European Atlantic coast^33,39^. Particularly, erosion of the small dune cordon and overwash in January 2014 was followed by man-made reprofiling and consolidation of the dune-embankment at a more landward position.

### Field surveys

Table 1 lists the different field survey products obtained over the period 2003–2019 and contained in the complete archived dataset^52^.

**Table 1:** Summary of Porsmilin topographic and bathymetric survey program.

PORSMILIN BEACH SURVEY PROGRAM			
Survey technique	Survey period	Number of surveys^a^	Survey coverage
RTK-GNSS (GPS)	January 2003 - continuing	253	Central cross-shore profile (subaerial beach)
RTK-GNSS (GPS)	March - April 2004	6	Subaerial beach DEM
Terrestrial Laser Scanning (TLS)	June 2009 - continuing	36	Subaerial beach DEM
Unmanned Aerial Vehicle photogrammetry (PHO)	October 2010 - continuing	6	Subaerial beach DEM and orthoimage
Boat-mounted multi-beam echo-sounding (MES)	September 2008 - continuing	24	Subtidal beach DEM
Data fusion between topographic and bathymetric surveys (FUS)	June 2009 - continuing	11^b^ 14^c^	Subaerial and subtidal beach DEM Central cross-shore profile

^a^Number of surveys until end 2019. ^b^Data fusion between topographic and bathymetric DEMs. ^c^Data fusion between measured beach profiles and DEM-extracted bathymetric profiles allowed us to increase the number of data fusions possible (cf. Fig. 2d).

**Fig. 2:**
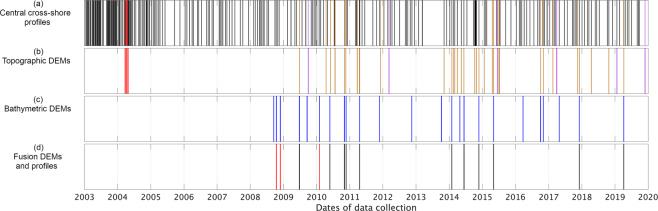
Survey coverage versus time. (**a**) Central cross-shore profiles (subaerial beach). Black strips indicate measured beach profiles using the GPS survey method, other colours are DEM-extracted profiles. (**b**) Topographic DEMs collected using RTK-GNSS (‘GPS’) (red), terrestrial laser-scanning (‘TLS’) (brown) and UAV photogrammetry (‘PHO’) (purple). (**c**) Multibeam echo-sounding (‘MES’) bathymetric DEMs. (**d**) Data fusion between measured topographic profiles and DEM-extracted bathymetric profiles (red) and data fusion between topographic and bathymetric DEMs (black).

Beach profile surveys consists in measuring positions along a central cross-shore profile, following a line from the dune-embankment down to the low-tide waterline. Positions are recorded using high-accuracy RTK-GNSS (simply named ‘GPS’ in the dataset). Topographic surveys are timed to coincide with low tides. Survey frequency was approximately weekly for the first year then fortnightly between January 2004 and June 2005. Thereafter, survey frequency was reduced to a more sustainable near-monthly approach, although gaps in the data eventually occurred, such as in 2013 (Fig. 2a). Daily surveys over approximately one-week periods were also carried out at a few instances during the course of the program (e.g., in April 2011 and October 2014). The cross-shore spacing between survey points is adapted to the terrain morphology, ranging from near-continuous (i.e., approximately 0.1 m) where it is complex, up to several metres in morphologically simple areas with homogeneous terrain slope. As is common practice, measured cross-shore profiles initially formatted as three-column x (easting), y (northing), z (elevation) matrices were projected onto the best-fit transect line determined by least-squares (y = 2.072 x + 6560.283, Pearson R = 0.99, n = 253) to be expressed in terms of vertical elevation versus horizontal cross-shore distance from the profile head (chainage). In 2014, the profile head was moved to a new position approximately 19 m landward, to adjust to the retreating dune position, without changing the orientation of the surveyed line (cf. Table 2). For all profiles, a zero chainage corresponds to this new profile head location.

**Table 2:** Dataset—subaerial and subtidal cross-shore profiles.

BEACH PROFILE DATASET				
Folder	Origin and orientation [Lat/Long/degN]	Data File	Time-series	File format
Profile_topo	48°21’21.946” N 4°40’46.173” W 205.763°	*yyyymmdd*_Porsmilin_GPS_Dist_IGN69_0.5_P.txt	2003–2019	Column 1 – Chainage (m) Column 2 – Elevation (m NGF)
		*yyyymmdd*_Porsmilin_*SurveyMethod*_Dist_IGN69_0.5_DP.txt		
Profile_bathy	Same as above	*yyyymmdd*_Porsmilin_MES_Dist_IGN69_0.5_DP.txt	2008–2019	Same as above
Profile_fusion	Same as above	*yyyymmdd*_Porsmilin_FUS_Dist_IGN69_0.5_DP.txt	2008–2019	Same as above

Elevations are relative to the national elevation datum NGF-IGN69 (m NGF), corresponding to approximately 0.5 m below MSL. Orientation of beach profiles is expressed in degrees North, whereby 180 degN indicates South counted clockwise. Lat/Long are referenced to WGS84.

To allow GPS surveys, a landmark serving as a base-station setting point was materialized atop the beach and geodetic survey marks installed on stable ground (e.g., rock outcrops and human structures) for accuracy verification (refer ‘Technical Validation’). Coordinates used for the dataset are metric and referenced to the legal coordinate system for metropolitan France, i.e., RGF93-Lambert 93 horizontal and NGF-IGN69 vertical (EPSG:5698). Elevation zero (m NGF) corresponds to approximately 0.5 m below mean sea level (MSL).

In addition to line surveys along the central cross-shore profile, surveys of the whole beach have also been undertaken. Between March and April 2004, a total of six 3D point clouds (Fig. 2b) of the subaerial beach excluding the reef, corresponding to surveyed areas between 15,000 and 30,000 m^2^ (cf. Online-only Table 1), were obtained using the GPS survey method.

From 2009 onwards, time-of-flight terrestrial laser-scanning (survey method ‘TLS’) and unmanned aerial vehicle (UAV) photogrammetry (survey method ‘PHO’) were progressively implemented in parallel to the on-going profile surveys, with the intention to better capture geomorphic changes and underlying processes through increasing coverage and spatial resolution. Particularly, photogrammetry and laser-scanning made possible the efficient measurement of the small dune-embankment, intertidal reef and cliffs.

TLS and PHO surveys start with the measurement of ground control points (GCPs) using RTK-GNSS for data georeferencing and verification. TLS scans are collected at 360° horizontally from different locations (called stations) to achieve uniformly high resolution across the beachface and to reduce occlusions. Typically, two stations were enough providing they were correctly positioned cross-shore centrally on the beach. With such configuration, much of the subaerial beach is no further than 100 m from the laser head. A Riegl LMS-Z390i laser-scanner with a vertical scanning range of 80° and an angular resolution of 0.07° was used until 2014, progressively replaced by a VZ-400 of the same manufacturer allowing even smaller angular resolution at 0.04° and a vertical scanning range of 100°. In either case, this corresponds to a maximum ground sampling distance of approximately 0.1 m at a distance of 100 m. Obtained point clouds are processed using the laser proprietary software RiSCAN PRO v1.7-2.0 for scan registration and geo-referencing (http://www.riegl.com/products/software-packages/riscan-pro/), and CloudCompare (http://www.cloudcompare.org/) for data interpolation into a DEM. The manufacturer stated positional accuracy and precision for the VZ-400 (LMS-Z390i) is 5(6) and 3(4) mm, respectively. This is an order of magnitude below the 3D root-mean square errors (RMSE) we estimated using GCP coordinates during processing, which yield overall scan precision of 0.026 ± 0.01 m ($\mu \pm 1\sigma$, n = 74, cf. Online-only Table 2).

Drone imagery and GCPs have been processed using the popular Structure-from-Motion (SfM) method implemented in Agisoft Photoscan (now Metashape; https://www.agisoft.com/), to produce geo-referenced DEMs and orthophotographs of the subaerial beach (Online-only Table 3 & 4). Different UAVs and optics have been used since the first photogrammetric surveys in 2006, starting with custom-built helicopter and multi-rotor drones equipped with a digital single lens reflex (DSLR) camera^40,41^. Profiting from the recent advent of high-quality commercial drones, drone surveys are now assured by a DJI Phantom 4 Pro and Phantom 4 RTK. Flying heights are typically around 80 m (maximum of 110 m), translating to a maximum ground pixel size of 0.02 m. Using some GCPs as check points (ChkPts) shows an overall photogrammetric 3D precision (RMSE) to be within the range 0.02 – 0.1 m (*μ* = 0.044 m, *σ* = 0.037 m, n = 6, cf. Online-only Table 3). Photogrammetric surveys before 2014 were initially designed to be processed using traditional stereo-photogrammetric workflows^40^. To be consistent with other surveys, these datasets were later reprocessed using the SfM method.

In total, 24 bathymetric DEMs have been obtained over the period 2008–2019 from hydrographic surveys using a boat-mounted multi-beam echo sounder (survey method ‘MES’, Online-only Table 5). Bathymetric surveys are generally undertaken during spring high tides to achieve good coverage of the upper shoreface. A topographic survey (DEM or profile) is often planned the same day at low tide (Fig. 2), providing independent data for quality verification and data fusion. Fusion DEMs and profiles (survey method ‘FUS’) are seamless subaerial and subtidal DEMs and transect lines obtained through averaging elevations at the overlap between concurrent topographic and bathymetric surveys (cf. Online-only Table 6 & 7). The hydrographic equipment aboard the ship consists in a multi-beam echo sounder (RESON SeaBat 8160 until the end of 2011, KONGSBERG EM 3002 thereafter) connected to RTK-GNSS and inertial measurement unit sensors. In addition, one or multiple sound speed profiles of the water column are acquired during the survey using a dedicated celerity probe. Using the EM 3002 (similar specifications prevail for the SeaBat), data are recorded by 254 equidistant beams emitting sound pulses at 300 kHz. Angular resolution is set to 120°, resulting in a maximum lateral coverage of approximately 50 m per track and inter-beam distances of 0.2 m at 15 m water depth (the maximum depth at the study area). Planning, collection and processing of bathymetric data are done using QPS Qinsy software suite (https://qps.nl/qinsy/), which after guided correction of inertial movements of the boat, sound celerity gradients and tidal effects, allows producing bathymetric DEMs with sub-metre spatial resolutions and theoretical accuracies/precisions ~0.1 m.

Using quality-controlled DEMs (cf. explanation below), elevation transects were systematically extracted corresponding to the central cross-shore profile line, hence producing bathymetric and fusion profiles, and increasing the number of subaerial beach profiles for the period 2003–2019 from initially 253 to 280 (Table 1, Fig. 2a).

### Data harmonization

**Fig. 3:**
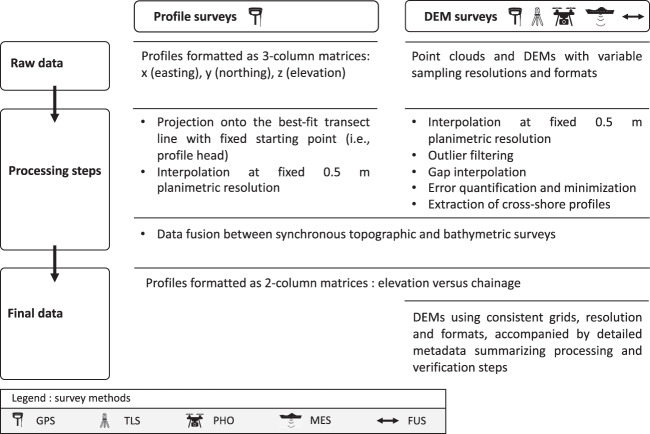
Workflow used for preparing the final dataset.

Figure 3 summarizes the key steps for preparing the final dataset. Starting in 2019, an important effort was devoted to revamp the dataset, particularly DEMs, which were obtained using different survey methods, in order to produce a suite of consistent observations easily comprehensible and reusable.

To facilitate reuse of the data and to allow direct comparisons, a standard sampling resolution of 0.5 m and consistent DEM grids were chosen to fit the entire dataset. Using a grid spacing of 0.5 m preserved high data quality, particularly for photogrammetry and laser-scanning allowing higher resolutions, while maintaining file size small for efficient handling. Consequently, some surveys were resampled (using linear interpolation) to a resolution higher than their initial sampling distances. This is the case of nine bathymetric surveys until end 2011 initially produced at 1 m and the six GPS point clouds obtained in 2004. Although small-scale bed features (<1 m) may not be detectable using extrapolated DEMs, the negative impact of up-sampling a small portion of the DEMs over the duration of the dataset on change detection analyses (e.g., sand budgeting) is assumed to be minimal at the beach scale.

To reduce operators’ influence on decisions such as filtering outliers and filling gaps in initial data, all DEMs were applied the same post-processing routine that consists in (i) using the mean elevation difference parameter to filter extreme deviations^53,54^ (here DEM elevations outside the range $\mu +5\sigma$, with *μ* and *σ* calculated for each surface grid cell using a three-by-three moving window), and (ii) reasonably filling missing data by averaging at least three known elevations. For the latter, search is done within circles centred on the missing DEM cell with radii increasing incrementally (increment = grid spacing) until at least three known values are found or until a size limit is attained (1 and 10 m over reef and sand, respectively). Reef/sand classification was performed using a combination of orthoimage and roughness analyses. The results of post-processing are reported in the metadata accompanying each DEM and are summarized in Online-only tables.

As a final step, all DEMs were assessed for systematic and random errors, with eventual corrections applied (cf. ‘Technical Validation’). Doing so enabled for the provision of total error estimates for each survey and ensured altimetric consistency throughout the duration of the monitoring program, these two steps being important prerequisites for allowing reliable change detection using long-term morphological datasets^55^.

### Waves

Continuous hourly wave characteristics directly offshore of Porsmilin in approximately 15 m water depth are provided for the period January 2000-December 2019. The wave dataset starts three years before the field surveys to provide antecedent conditions that may be useful for understanding and modelling beach state at the start of the field surveys^22,56^. Wave parameters provided are the significant wave height (Hs), peak (Tp) and mean (T02) wave periods, and peak wave direction (Dir). They were derived from sea-states hindcast databases developed by Ifremer, using the node point 47554 (48°20'47.76” N, 4°40’52.32” W, Fig. 1). Databases are based on Wavewatch III (WW3) model (version 4.11) using the same unstructured grid covering the English Channel and Bay of Biscay with a resolution at the coast of 200 m. The first database called HOMERE is a global hindcast produced in 2017 and covering the period 1994–2016 (23 years)^57^. The second database, NORGAS-UG, is updated monthly with archived data that currently spans 2008–2019^58^. Wave parameters from the two databases were combined to produce the wave dataset over the period 2000–2019 (cf. ‘Technical Validation’).

### Tides

Continuous tidal levels at 10-min intervals are provided over the same time period as the combined wave dataset (i.e., January 2000-December 2019). They were derived using the tidal analysis package for MATLAB Utide^59^ based on hourly observed water-level data^60^ (22/12/1970–31/12/2019) at the nearby Shom (Service hydrographique et océanographique de la marine) tide gauge of Le Conquet (48°21'32.753“N, 4°46’50.7”W, Fig. 1b), situated less than 8 km from the field site.

## Data Records

The archived datasets presented herein, spanning 2003–2019 for field surveys and 2000–2019 for waves and tides can be accessed at Indigeo^52^. With the monitoring program continuing, we intend to update the data on an annual basis through DOI versioning. Field survey products as they continue to be updated can also be accessed and visualised at https://www.dynalit.fr/La-carte-des-sites/Porsmilin#/map.

Table 2 documents the repository folders, data format and metadata for the subaerial and subtidal cross-shore profile dataset. Profiles originate either from the GPS profile surveys (labelled with ‘_P’ in that instance) or from DEM surveys (‘_DP’). For the latter, the survey method can be either ‘GPS’, ‘TLS’, ‘PHO’, ‘MES’ or ‘FUS’ depending on how the DEM was obtained. Profiles come as individual tabulation-delimited text files (.txt) with the date and survey method provided in the title. Each profile is expressed in terms of vertical elevation versus cross-shore distance from the profile head (chainage) at a standard spacing of 0.5 m.

**Fig. 4:**
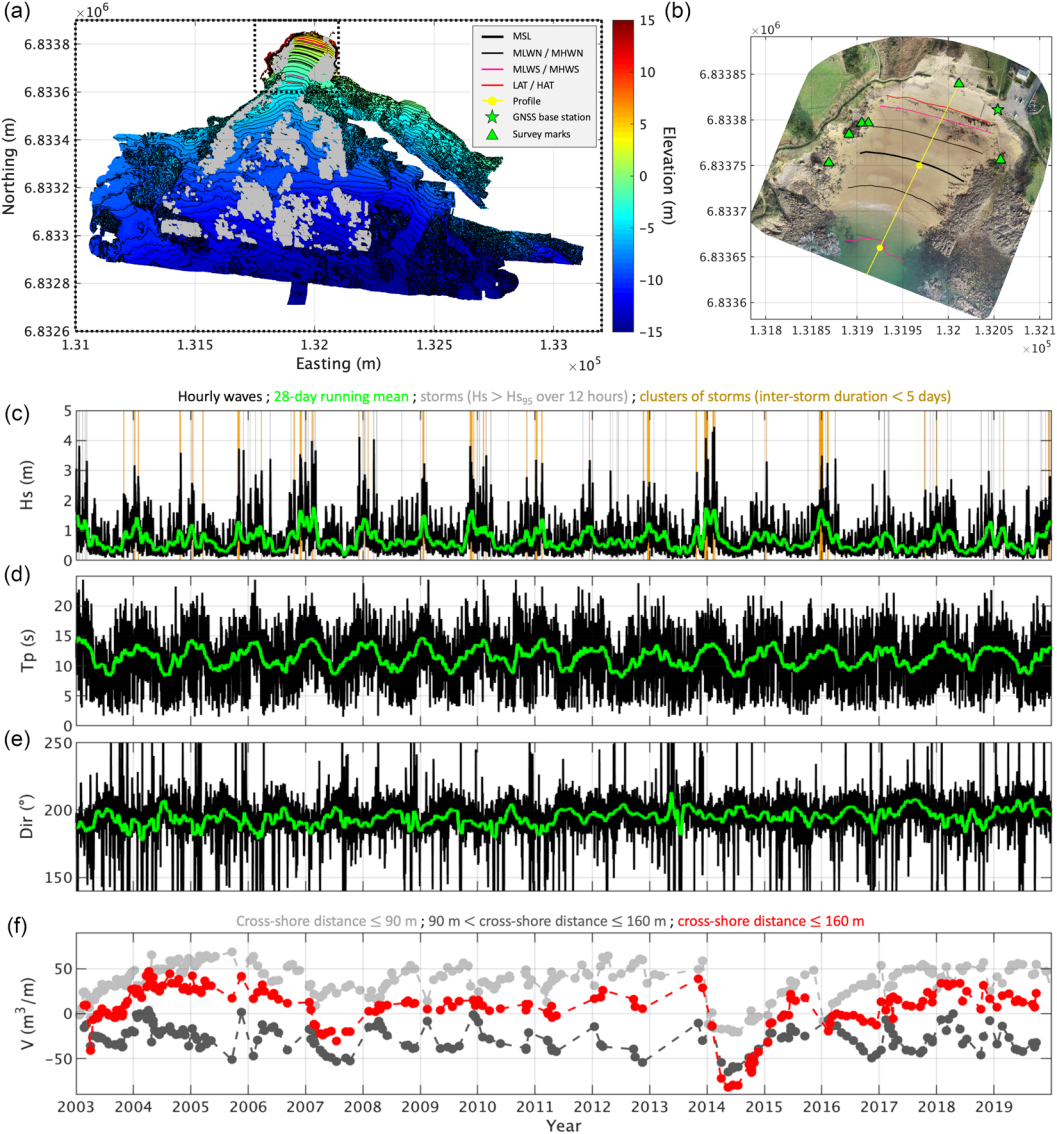
Example of spatial and time-series products. (**a**) Composite DEM at 0.5 m resolution, formed by averaging all fusion DEMs, showing elevation contours at 1 m intervals and specific tidal levels. Dashed rectangles correspond to respective grid coverages for topographic and bathymetric DEMs. Greyed areas correspond to ground truth elevations used for DEM quality assessment (n > 100,000). (**b**) Orthoimage at 0.1 m resolution showing the central cross-shore profile line with graduations every 100 m (yellow), the location of the RTK-GNSS base station (green star) and geodetic survey marks used for profile verification (green triangles). (**c**,**d**,**e**) Hourly (black) and 28-day running mean (green) estimates of inshore (node 47554) significant wave height (Hs), peak wave period (Tp) and peak wave direction (Dir), respectively. Grey and orange bars in (**c**) show significant storms (i.e., Hs exceeding Hs_95_ over at least 12 hours, where Hs_95_ is the wave height that is exceeded only 5% of the time over a one-year period) and clusters of storms (i.e., successive storms with inter-storm duration less than 5 days), respectively. (**f**) Sand volumes per metre of alongshore beach length estimated using measured and DEM-extracted profiles over the upper intertidal beach (chainage $\le$ 90 m, light grey), lower intertidal beach (chainage = 90-160 m, dark grey) and complete beach face (chainage $\le$ 160 m, red).

Table 3 documents the repository folders, data format and associated files for the subaerial and subtidal DEM dataset. DEMs are provided on a regular grid with 0.5 m spacing and surface coverage (easting by northing) of 350 × 300 m^2^ for the topographic DEMs and 2200 × 1300 m^2^ for bathymetric and fusion DEMs (cf. Figure 4a). Each DEM comes in a separate folder containing the DEM in ArcGrid ASCII (.asc), geotiff (.tif) and MATLAB data (.mat) formats. Unlike .asc and .tif standard export formats for geographic datasets^21,61^, which were chosen because of their size efficiency and the ability to easily incorporate them into a (web) GIS, .mat files have the ability to store additional information. Here, it includes the source DEM or point cloud, a quantitative description of post-processing steps (‘filter’ and ‘fill’, providing the location of filtered and interpolated cells) and the results of data verification (‘z_georef_check’). Accompanying each DEM is a detailed metadata text file that summarizes the results of the main post-processing and validation steps. DEM folders also contain an image imprint of the DEM, as well as a DEM of Difference (DOD) and a figure plot of the associated Probability Density Function (PDF) of DEM errors obtained after comparison with a ground truth limited to the reef (cf. ‘Technical Validation’), all in Portable Network Graphics (.png) format. Because GPS DEMs are restrained to sand only (the surrounding reef was not measured), they could not be compared to available ground truths, and hence DOD and PDF figure plots are not included. For fusion DEMs, DOD and PDF figure plots are based on the comparison between overlapping topographic and bathymetric data. Photogrammetric DEMs are accompanied by orthophotos obtained concurrently during processing, provided in geotiff at a resolution of 0.1 m for improved usability. Orthophotos enabled for the quantitative assessment of planimetric errors (shift, rotation and scale) resulting from photogrammetric surveys and processing methods, which are reported in the metadata text files provided.

**Table 3:** Dataset-subaerial and subtidal DEM surveys.

DEM SURVEY DATASET			
Folder	Areal extent (m^2^)	Data Files	Time-series
DEM_topo	350 × 300	*yyyymmdd*_Porsmilin_*SurveyMethod*_L93_IGN69_0.5.asc	2004–2019
		*yyyymmdd*_Porsmilin_*SurveyMethod*_L93_IGN69_0.5.tif	
		*yyyymmdd*_Porsmilin_*SurveyMethod*_L93_IGN69_0.5.mat	
		*yyyymmdd*_Porsmilin_*SurveyMethod*_L93_IGN69_0.5_MetaData.txt	
		*yyyymmdd*_Porsmilin_*SurveyMethod*_L93_IGN69_0.5.png	
		*yyyymmdd*_Porsmilin_*SurveyMethod*_L93_IGN69_0.5_DOD.png	
		*yyyymmdd*_Porsmilin_*SurveyMethod*_L93_IGN69_0.5_PDF_error.png	
DEM_bathy	2200 × 1300	Same as above	2008–2019
DEM_fusion	2200 × 1300	Same as above	2009–2019

Horizontal and vertical coordinates in DEMs are relative to the French metropolitan coordinate system RGF93-Lambert 93 and national elevation datum NGF-IGN69, respectively.

Table 4 documents the format and metadata for the hourly time-series of inshore significant wave height (Hs), peak (Tp) and mean (T02) wave periods, and peak wave direction (Dir), estimated in approximately 15 m water depth directly offshore of Porsmilin beach (node point 47554, Fig. 1c). The tabulation-delimited file is called ‘Porsmilin_wave.txt’.

**Table 4:** Dataset-Inshore Waves.

HINDCAST WAVE DATASET			
Location [Lat/Long]	Data File	Time-series	File format
48°20’47.76” N 4°40’52.32” W	Porsmilin_wave.txt	01/2000-12/2019 hourly	Columns 1 to 4 – Date and time (CET, yyyy,mm,dd,HH) Column 5 – Significant wave height Hs (m) Column 6 – Mean wave period T02 (s) Column 7– Peak wave period Tp (s) Column 8 – Peak wave direction Dir (degN)

Time is relative to 24 h Central European Time – CET. Lat/Long are referenced to WGS84.

Table 5 documents the tabulation-delimited text file called ‘Porsmilin_tide.txt’, format and metadata for the time-series of tide levels sampled every 10 minutes and spanning the identical period of the inshore wave time-series.

**Table 5:** Dataset-Astronomical tides.

ASTRONOMICAL TIDE DATASET			
Location [Lat/Long]	Data File	Time-series	File format
48°21’32.753” N 4°46’50.700” W	Porsmilin_tide.txt	01/2000-12/2019 10 min	Columns 1 to 5 – Date and time (CET, yyyy,mm,dd,HH,MM) Column 2 – Astronomical tide (m CD) Column 3 – Astronomical tide (m NGF)

Time is relative to 24 h Central European Time – CET. Lat/Long are referenced to WGS84. Tidal levels are expressed both with reference to the chart datum (i.e., the tide gauge reference corresponding to the lowest astronomical tides) and to the national elevation datum NGF-IGN69.

## Technical Validation

### Profile surveys

Measured beach profiles were systematically validated using geodetic survey marks. There are now five survey marks distributed across the study site materialized by cast metal disks sunk into bedrock and an additional survey mark affixed to the top of a pipe sunk into the dune-embankment and materializing the profile head (Fig. 4b). Re-analysis of survey marks’ coordinates indicates GPS precisions (estimated as one standard deviation) along x (easting), y (northing) and z (elevation) of 0.012, 0.015 and 0.035 m, respectively, which is close to the maximum precision achievable using this survey method^62^.

### DEM surveys

The proportion of surface cells considered outliers represented no more than five permille, considering all topographic and bathymetric DEMs (*μ* = 3.6 permille, *σ* = 0.8, n = 72), with little differences between survey methods. The proportion of cells that were interpolated amounted to less than 16 permille on average over sandy sections (*μ* = 15.5 permille, *σ* = 27.8, n = 72). Disparities between surveys exist, but there is no clear relation with the survey method used. Small gaps in coverage of sandy sections can be due to different reasons, including spurious elevations most often due to people on the beach, that were filtered, light or laser reflections on wet surfaces, insufficient image texture locally preventing effective pixel matching for photogrammetry, and incomplete boat tracks due to obstacles, waves and time running short. Although surveys are designed to limit gaps, they cannot be avoided entirely. A sensitivity analysis (not shown) of the filling method was conducted by varying the maximum radius for search and the minimum numbers of known values for interpolating a missing DEM cell. The analysis was performed on a composite DEM (Fig. 4a), to which gaps of variable size provided from other DEMs were artificially created (resulting in the deletion of approximately 12,000 cells). Using a maximum radius for search of 10 m and a minimum number of known elevations set to three enabled to fill most data gaps ( >99%) without impacting the reliability of interpolated sand levels (mean error (ME) and standard deviation of error (SDE) ~10^−6^ and 0.01 m, respectively). Importantly, this traduced to a sand volume error of only 0.4 m^3^ over the complete DEM size, which is several orders of magnitude below the uncertainty that would be associated to a typical beach survey precision of 0.1 m.

In line with recent studies advocating for quantitative statements regarding data quality, to ensure reliable morphological analyses using coastal topographic data^28^, technical validation of this dataset was possible using a variety of error statistics adapted to the survey methods and all reported in this data descriptor. Errors estimated at intermediate stages of the dataset preparation (e.g., RMSE reported by photogrammetry and laser-scanning software) can sometimes be misleading, as they may not include all possible error sources, and hence cannot be used confidently to ascertain consistency between topographic and bathymetric products forming this long-term dataset.

Final DEM quality was assessed in terms of accuracy, precision and reliability through fit-for-purpose experiments. Accuracy or bias, reported as the mean error, and survey precision, reported as the standard deviation of error, were estimated in comparison with a reference DEM (ground truth) comprising the most stable parts of the study site (cf. explanation below). Using the comparison with a ground truth also enabled to detect eventual tilts (i.e., out-of-plane rotations) in DEMs by fitting a linear surface by least-squares to elevation residuals (DOD). The locations where the data overlapped between topographic and bathymetric surveys provided a final means of confirming the reliability of the data.

For ground truths, we used composite DEMs limited to reef obtained by averaging all available DEMs iteratively, such that only the most consistent DEMs (corresponding to approximately 25% rejection after statistical testing at one standard deviation on bias and precision) and surface cells with high representation (i.e., cells estimated using at least two-third of available DEMs) were retained. The bathymetric ground truth counted over 130,000 known elevations, computed using 19 independent measurements (out of 24), with at-a-cell vertical precision of 0.07 m (determined as one standard deviation between independent measurements, averaged over all cells). Similar results were obtained for the topographic counterpart, but due to the large difference in area covered, the ground truth accounted for just over 10,000 truth elevations (cf. Table 6). The at-a-cell vertical precision is a good proxy for the overall precision of the dataset, as it characterises the ability to effectively replicate measurements over time.

**Table 6:** Validation of the DEM dataset for the different survey methods.

DEM VALIDATION					
Survey method	MES	GPS	TLS	PHO	FUS
**Bias**	0.041 ± 0.028 m	0.003 ± 0.000 m	0.039 ± 0.039 m	0.034 ± 0.028 m	0.040 ± 0.039 m
**Precision**	0.071 ± 0.068 m	0.033 ± 0.011 m	0.077 ± 0.030 m	0.110 ± 0.091 m	0.063 ± 0.025 m
**Number of validation points**	102057 ± 39064	279 ± 88	8542 ± 2415	11190 ± 5090	13781 ± 10974

All values are presented as $\mu \pm 1\sigma$, determined using all DEMs of the same method. Individual error statistics from which these values are derived are presented in Online-only tables. Bias is the mean deviation (calculated either as ‘measurement – ground truth’ or ‘bathymetry – topography’) expressed in absolute value, thus representing the actual magnitude data can be offset from a reference level, regardless of the direction.

Using the ground truths, DEMs that initially presented a vertical bias (>0.1 m) but high precision, suggesting a systematic and easily rectifiable shift in elevation, generally owing to improper setting of the vertical datum, were corrected, effectively reducing registration errors across the dataset (Table 6). DEMs identified as presenting large and complex errors (e.g., tilts) are not included in the dataset. This essentially concerns photogrammetric DEMs collected prior 2014. Final error statistics are provided in the metadata accompanying DEMs and are presented in Online-only tables for this paper. The evaluation shows that all survey methods were able to produce high quality data, shown by bias and precision mostly below 0.1 m. No clear degradation of data quality resulting from measuring submerged topographies can be observed. Note that because GPS DEMs are limited to sand, where morphological changes occur, this prevented us from using the data validation procedure presented above. Where possible, a GPS DEM was compared with a synchronous (i.e., same day) cross-shore profile, also providing information on mean deviations, precision and tilt.

### Waves

The continuous hourly wave dataset for Porsmilin is comprised of HOMERE (01/2000 – 12/2016) and NORGAS-UG (01/2017-12/2019). To avoid discontinuities in the time-series, NORGAS-UG data were corrected for bias, based on the comparison between HOMERE and NORGAS-UG at the node point 47554 (Table 7).

**Table 7:** Validation of HOMERE and NORGAS-UG wave hindcast datasets.

WAVE HINDCAST VALIDATION														
Model	Node	n	Hs			Tp			T02			Dir		
			R	Bias	rms	R	Bias	rms	R	Bias	rms	R	Bias	rms
HOMERE	47039	75,358	0.98	0.17 m	0.32 m	0.79	−1.37 s	2.04 s	0.89	−0.20 s	0.80 s	0.52	−1.56°	28.41°
NORGAS-UG	47039	96,273	0.97	0.16 m	0.35 m	0.76	−1.70 s	2.34 s	0.89	−0.49 s	0.89 s	0.56	−0.88°	27.92°
HOMERE	47562	976	0.97	0.02 m	0.11 m	0.62	−1.32 s	2.42 s	0.36	−0.37 s	2.11 s	0.30	−2.16°	10.65°
Intercomparison	47562	78,912	0.97	0.03 m	0.13 m	0.81	−0.45 s	1.91 s	0.82	−0.70 s	1.46 s	0.68	0.74°	10.45°
Intercomparison	47039	78,912	0.98	5e^−3^ m	0.24 m	0.90	−0.24 s	1.05 s	0.95	−0.27 s	0.59 s	0.78	0.30°	19.81°
Intercomparison	47554	78,912	0.97	0.04 m	0.13 m	0.80	−0.46 s	1.98 s	0.82	−0.66 s	1.42 s	0.70	7e^−3^°	14.14°

The first three lines correspond to model-buoy comparisons. CANDHIS wave buoys at Pierres Noires and Porsmilin correspond to hindcast nodes 47039 and 47562, respectively. The bottom three lines correspond to comparisons between HOMERE and NORGAS-UG over the common period of hindcast.

As demonstrated in previous work, HOMERE and NORGAS-UG have been validated at both global and regional levels using a combination of *in-situ* measurements, remote sensing from satellite altimeters and outputs from the NOAA/NCEP configuration of WW3^63,64^. We further assessed the capability of these wave hindcasts to provide realistic offshore and inshore conditions for Porsmilin by comparing hindcast data at the nearest grid point to available wave buoy measurements (http://candhis.cetmef.developpement-durable.gouv.fr/) at Pierres Noires and Porsmilin (WW3 nodes 47039 and 47562, respectively). The Pierres Noires buoy (Fig. 1b,d) has recorded bi-hourly wave estimates in 60 m water depth since October 2005. Between March and April 2004 (period not covered by NORGAS-UG), a directional wave buoy was installed temporarily offshore Porsmilin (Fig. 1c). Intercomparing outputs from HOMERE and NORGAS-UG taken separately enabled assessment of the models’ reliability over the common period of hindcast (i.e., 01/2008-12/2016, n = 78,912).

The results of the validation for all four wave parameters (Hs, Tp, T02 and Dir) at both offshore and inshore locations, using the Pearson correlation coefficient R, mean bias (estimated as ‘hindcast – measured data’ for model-buoy comparisons and ‘NORGAS-UG – HOMERE’ for model inter-comparisons) and RMSE, are presented in Table 7. HOMERE and NORGAS-UG both demonstrate overall excellent skill modelling offshore and inshore waves, particularly for Hs, with R values constantly above 0.97. Agreement with *in-situ* data decreases slightly for Tp, T02 and Dir. The latter was observed in previous research^22,63,64^ and explained by the differences in accounting for mixed sea states (i.e., the superimposition of different wave periods and/or directions) by wave buoys and hindcast, respectively.

### Tides

Water levels are based on validated tidal gauge measurements provided by Shom, the French authority regarding tidal model development and tidal observation, operating over 30 stations across metropolitan France^60^. Gaps in hourly measured tidal levels over the period 1970-2019 represented 3.6% of the data.

## Usage Notes

### Field surveys

Using the dataset, subaerial sediment dynamics from event to pluri-annual time scales can be assessed through sediment budget analyses, complemented since 2008 by approximately bi-annual bathymetric surveys capturing sediment transfers with the shoreface. Due to relocation of the profile head in 2014, profile comparisons are best performed by excluding the first 20 meters or so, as this section of the beach was not measured before 2014. Likewise, quantification of back beach and foredune-embankment changes will benefit from analysing separately the surveys undertaken before and after relocation of the profile head.

DEMs provide very detailed maps of beach morphology and allow for numerous cross-shore and alongshore profiles to be extracted. DEMs are provided on a constant grid making for easy computations of temporal changes without requiring intermediate steps, while format selection ensures that DEMs are readily usable with a variety of GIS and scientific programming software. Error estimates provided in the metadata are directly usable to feed uncertainty-based geomorphic change detection analyses, as the reliability of findings can be greatly improved by correctly accounting for measurement uncertainty^55^.

### Waves

Hindcasted wave parameters were validated using both offshore and inshore wave buoys, showing good agreement, particularly for Hs. However, careful analysis of models’ comparisons with the Pierres Noires wave buoy (offshore buoy), for which observations cover several winters, suggests that the most energetic events may not be captured as well by the models, potentially resulting in reduced wave heights in comparison to buoy readings (sometimes up to 10% reduction). This effect was observed in previous research^63,64^. It can be explained, in decreasing order of importance, by the accuracy of the forcing fields (e.g., wind and current) and the behaviour of the physical parameterizations, which may be particularly sensitive during storms. Although the Porsmilin wave buoy (inshore buoy) captured wave heights above 2 m, no reduction in modelled wave heights was observed. The validity of this finding may be hindered however by the short period of observation.

### Water levels

All significant tidal levels can be accessed at ref. ^51^ (p. 30). They can be used to transform the vertical datum used in this study (m NGF) to have a zero-mean equal to mean sea level, by subtracting 0.52 m. Water levels provided here do not include surges at the coast, which are the result of a combination of meteorological and sea-state factors not measured, and which may be important during storms. Surges can be evaluated using coastal models hindcasts (http://marc.ifremer.fr/en/resultats/niveaux). Likewise, analysis of shoreline water levels requires computing the total run up at the shore from the wave hindcasts provided and adding tidal levels, which may be done using equations presented in ref. ^65^.

### Complementary datasets

A seamless subaerial and subtidal LiDAR DEM^66^ obtained between 2011 and 2013 can be accessed at https://diffusion.shom.fr/pro/risques/litto3dr-finistere-2014.html. Wave data for other hindcast nodes can be accessed at 10.12770/cf47e08d-1455-4254-955e-d66225c9dc90 (HOMERE^57^) and https://sextant.ifremer.fr/record/0873e969-6c97-4405-a040-fd4599f5c936 (NORGAS-UG^58^). Additional tide data from Shom^60^ can be accessed at 10.17183/REFMAR.

## Data Availability

Dataset preparation and validation was performed using MATLAB (R2018b). Computer programs are included in the dataset. They can be used for browsing through the dataset and plotting some of the results. Although MATLAB is a proprietary language, the.m files can be read with a text viewer.

## Online-only Tables

**Online-only Table 1:** GPS DEMs.

	General information				Data collection			DEM processing				DEM validation			
	Name of DEM file	Date	Tidal coeff.	Beach coverage	Instrument	Number of survey points	Mean point spacing	Final grid spacing	Area	Number of filtered DEM cells	Number of filled DEM cells	ME	SDE	Tilt	Profile length where data overlap
							(m)	(m)	(m^2^)			(m)	(m)	(deg)	(m)
1	20040322_Porsmilin_GPS_L93_IGN69_0.5	3/22/2004	99	MLWS ->	Trimble 5800	310	4.4	0.5	26910	377	450	−0.003	0.025	0	170
2	20040326_Porsmilin_GPS_L93_IGN69_0.5	3/26/2004	65	MLWN ->	Trimble 5800	410	3.8	0.5	23194	231	352	N.A.	N.A.	N.A.	N.A.
3	20040405_Porsmilin_GPS_L93_IGN69_0.5	4/5/2004	98	MLWS ->	Trimble 5800	543	3.8	0.5	30024	247	359	N.A.	N.A.	N.A.	N.A.
4	20040408_Porsmilin_GPS_L93_IGN69_0.5	4/8/2004	103	MLWS ->	Trimble 5800	327	4.1	0.5	24674	228	302	N.A.	N.A.	N.A.	N.A.
5	20040417_Porsmilin_GPS_L93_IGN69_0.5	4/17/2004	80	MLWN ->	Trimble 5800	306	4.3	0.5	23966	196	266	N.A.	N.A.	N.A.	N.A.
6	20040428_Porsmilin_GPS_L93_IGN69_0.5	4/28/2004	28	MSL ->	Trimble 5800	272	4.1	0.5	14877	81	145	0.003	0.04	0.03	108

**Online-only Table 2:** TLS DEMs.

	General information					Data collection					DEM processing					DEM validation				
	Name of DEM file	Date	Tide coeff	Beach coverage	Fusion	Instrument	Scan positions	Angular Resolution	Number of GCPs per scan	Standard deviation on GCPs	Initial grid spacing	Final grid spacing	Final area	Number of filtered DEM cells (all/sand)	Number of filled DEM cells (all/sand)	Vertical correction	ME	SDE	Tilt	Number of DEM cells used for validation
								(°)		(m)	(m)	(m)	(m^2^)			(m)	(m)	(m)	(deg)	
1	20090625_Porsmilin_TLS_LG3_IGN69_0.5	6/25/2009	97	MLWN ->	x	Z390i	9	0.2	N.A.	N.A.	0.1	0.5	40772	663/0	4172/1053		0.046	0.073	0.026	10257
2	20100415_Porsmilin_TLS_LG3_IGN69_0.5	4/15/2010	88	MLWN ->		Z390i	3	0.12	7	0.0388	0.1	0.5	24977	406/0	2256/197		0.027	0.106	0.051	6021
									7	0.0309										
									8	0.0380										
3	20100603_Porsmilin_TLS_LG3_IGN69_0.5	6/3/2010	51	MLWN ->	x	Z390i	3	0.07	8	0.0173	0.1	0.5	30136	533/0	3563/299	0.15	0	0.067	0.005	8775
									7	0.0123										
									7	0.0148										
4	20100722_Porsmilin_TLS_LG3_IGN69_0.5	7/22/2010	48	MLWN ->		Z390i	2	0.07	14	0.0297	0.1	0.5	28514	451/0	2036/66	0.224	0	0.171	0.06	7357
									9	0.0258										
5	20100723_Porsmilin_TLS_LG3_IGN69_0.5	7/23/2010	55	MLWN ->		Z390i	2	0.07	10	0.0266	0.1	0.5	32927	570/0	2563/12	0.927	0	0.13	0.04	9909
									14	0.0212										
6	20101104_Porsmilin_TLS_LG3_IGN69_0.5	11/4/2010	85	MLWN ->	x	Z390i	2	0.07	18	0.0130	0.2	0.5	30642	418/0	1387/180		0.064	0.097	0.046	9989
									12	0.0131										
7	20101118_Porsmilin_TLS_LG3_IGN69_0.5	11/18/2010	55	MSL ->	x	Z390i	2	0.07	10	0.0213	0.1	0.5	23972	407/0	777/0		0.004	0.097	0.017	6090
									10	0.0207										
8	20110323_Porsmilin_TLS_LG3_IGN69_0.5	3/23/2011	103	MLWN ->		Z390i	2	0.07	11	0.0125	0.1	0.5	31410	442/0	1296/189	0.108	0	0.07	0.017	8646
									5	0.0137										
9	20110324_Porsmilin_TLS_LG3_IGN69_0.5	3/24/2011	87	MLWN ->		Z390i	2	0.07	6	0.0701	0.1	0.5	29427	384/0	2411/1259		0.094	0.098	0.047	8452
									9	0.0809										
10	20110418_Porsmilin_TLS_LG3_IGN69_0.5	4/18/2011	109	MLWN ->		Z390i	2	0.07	8	0.0230	0.1	0.5	24134	347/0	1142/169		0.093	0.05	0.008	5254
									7	0.0386										
11	20110419_Porsmilin_TLS_LG3_IGN69_0.5	4/19/2011	111	MLWN ->		Z390i	2	0.07	16	0.1060	0.1	0.5	33079	469/0	2597/46		0.081	0.073	0.019	9688
									16	0.1025										
12	20110420_Porsmilin_TLS_LG3_IGN69_0.5	4/20/2011	106	MLWN ->	x	Z390i	2	0.07	4	0.0321	0.1	0.5	30685	442/0	3007/662	0.082	0.009	0.074	0.027	8586
								7	0.0957											
13	20111208_Porsmilin_TLS_LG3_IGN69_0.5	12/8/2011	62	MSL ->		Z390i	2	0.07	16	0.0156	0.5	0.5	32156	582/0	12245/809		-0.022	0.075	0.07	9437
									16	0.0156										
14	20131105_Porsmilin_TLS_LG3_IGN69_0.5	11/5/2013	100	MSL ->		Z390i	2	0.07	14	0.0129	0.5	0.5	38015	587/0	20866/6048	-0.099	0.03	0.075	0.022	9478
									18	0.0111										
15	20140130_Porsmilin_TLS_LG3_IGN69_0.5	1/30/2014	95	MLWN ->	x	Z390i	2	0.07	14	0.0162	0.1	0.5	38372	577/0	13959/1482	-0.084	0.023	0.059	0.035	8622
									14	0.0194										
16	20140218_Porsmilin_TLS_LG3_IGN69_0.5	2/18/2014	87	MLWN ->		Z390i	2	0.07	13	0.0128	0.5	0.5	37814	652/0	8325/2333	-0.134	0.043	0.061	0.007	9907
									12	0.0133										
17	20140303_Porsmilin_TLS_LG3_IGN69_0.5	3/3/2014	114	MLWN ->		Z390i	1	0.07	13	0.0144	0.5	0.5	29166	460/4	2766/1349	-0.116	0.028	0.057	0.013	8926
									12	0.0146										
18	20140331_Porsmilin_TLS_LG3_IGN69_0.5	3/31/2014	107	MLWN ->		VZ-400	2	0.04	15	0.0198	0.1	0.5	33426	472/0	9005/864	0.118	0.008	0.044	0.004	7212
									15	0.0223										
19	20140514_Porsmilin_TLS_LG3_IGN69_0.5	5/14/2014	86	MLWN ->		Z390i	2	0.07	13	0.0119	0.5	0.5	32290	390/3	2668/1422		-0.09	0.053	0.004	9947
									9	0.0107										
20	20140612_Porsmilin_TLS_LG3_IGN69_0.5	6/12/2014	82	MLWN ->	x	Z390i	2	0.07	16	0.0128	0.1	0.5	33968	560/3	17494/2175		-0.085	0.045	0.012	8215
									15	0.0136										
21	20141009_Porsmilin_TLS_LG3_IGN69_0.5	10/9/2014	110	MLWN ->		VZ-400	2	0.04	17	0.0270	0.5	0.5	40592	716/0	4027/663		-0.018	0.061	0.01	10212
									16	0.0236										
22	20141027_Porsmilin_TLS_LG3_IGN69_0.5	10/27/2014	86	MLWN ->		VZ-400	2	0.04	13	0.0235	0.5	0.5	0	586/0	2817/563		-0.101	0.052	0.018	9284
									12	0.0227										
23	20141124_Porsmilin_TLS_LG3_IGN69_0.5	11/24/2014	92	MLWN ->	x	Z390i	1	0.07	10	0.0141	0.1	0.5	35005	538/0	11860/1078		-0.077	0.076	0.008	7217
									9	0.0632										
24	20150122_Porsmilin_TLS_LG3_IGN69_0.5	1/22/2015	109	MLWN ->		VZ-400	2	0.04	16	0.0204	0.5	0.5	33390	538/0	7705/3886		-0.097	0.083	0.019	8848
									15	0.0235										
25	20150420_Porsmilin_TLS_LG3_IGN69_0.5	4/20/2015	110	MLWN ->		VZ-400	2	0.04	14	0.0204	0.5	0.5	37469	668/3	1862/627		-0.006	0.062	0.04	10121
									15	0.0179										
26	20150504_Porsmilin_TLS_LG3_IGN69_0.5	5/4/2015	84	MLWN ->	x	VZ-400	2	0.04	16	0.0248	0.5	0.5	38172	696/0	2517/279	-0.113	0	0.059	0.016	10272
									16	0.0227										
27	20150703_Porsmilin_TLS_LG3_IGN69_0.5	7/2/2015	85	MLWN ->		VZ-400	2	0.04	12 10	0.0150 0.0263	0.5	0.5	39384	651/0	1670/581		-0.06	0.09	0.018	10272
		7/3/2015	91				5	0.04	11 12 11 11 11	0.0108 0.0095 0.0140 0.0107 0.0110										
28	20150708_Porsmilin_TLS_LG3_IGN69_0.5	7/8/2015	73	MHWS ->		VZ-400	1	0.04	14	0.0304	0.5	0.5	10547	168/0	2474/588	-0.162	0	0.155	0.058	611
29	20161004_Porsmilin_TLS_LG3_IGN69_0.5	10/4/2016	82	MLWN ->		VZ-400	2	0.04	15	0.0264	0.5	0.5	31234	527/5	7000/1700	0.092	0	0.133	0.077	9354
									15	0.0315										
30	20161103_Porsmilin_TLS_LG3_IGN69_0.5	11/3/2016	76	MLWN ->		VZ-400	2	0.04	16	0.0200	0.5	0.5	38584	674/0	6671/687	-0.105	0	0.052	0.004	10253
									15	0.0179										
31	20170216_Porsmilin_TLS_LG3_IGN69_0.5	2/16/2017	73	MHWS ->		VZ-400	2	0.04	10	0.0257	0.5	0.5	7381	105/0	713/352		-0.066	0.037	N.A.	78
									14	0.0276										
32	20171116_Porsmilin_TLS_LG3_IGN69_0.5	11/16/2017	77	MSL ->		Z390i	1	0.07	12	0.0140	0.5	0.5	28763	514/0	3372/1615		-0.012	0.067	0.015	9355
33	20171205_Porsmilin_TLS_LG3_IGN69_0.5	12/5/2017	107	MLWN ->	x	VZ-400	2	0.04	14	0.0239	0.5	0.5	40605	703/0	9993/6563		0.033	0.065	0.014	10240
									14	0.0235										
34	20180416_Porsmilin_TLS_LG3_IGN69_0.5	4/16/2018	97	MHWS ->		VZ-400	1	0.04	8	0.0331	0.5	0.5	38140	669/0	13597/10903		0.092	0.067	0.013	10254
35	20181025_Porsmilin_TLS_LG3_IGN69_0.5	10/25/2018	94	MLWN ->		VZ-400	2	0.04	15	0.0237	0.5	0.5	38192	735/0	5910/2132		0.118	0.067	0.023	10215
									6	0.0267										
36	20190409_Porsmilin_TLS_LG3_IGN69_0.5	4/9/2019	83	MLWN ->	x	VZ-400	2	0.04	19	0.0119	0.5	0.5	34818	639/0	11718/7696	1.947	0	0.072	0.017	10165
									19	0.0197										

**Online-only Table 3:** PHO DEMs.

	General information				Data collection											DEM processing					DEM validation				
	Name of DEM file	Date	Tidal coeff.	Beach coverage	Drone	Camera	Altitude	Number of camera stations	Pixel pitch	Focal length	Ground pixel size	Number of GCPs	RMSE GCPs	Number of ChkPts	RMSE ChkPts	Initial grid spacing	Final grid spacing	Final area	Number of filtered DEM cells (all/sand)	Number of filled DEM cells (all/sand)	Vertical correction	ME	SDE	Tilt	Number of DEM cells used for validation
							(m)		(μm)	(mm)	(mm)		(m)		(m)	(m)	(m)	(m^2^)			(m)	(m)	(m)	(deg)	
1	20091001_Porsmilin_PHO_L93_IGN69_0.5	10/1/2009	60	MSL ->	DRELIO	Nikon D200	98	66	6.25	35	16.1	50	0.032	2	0.024	0.1	0.5	18974	243/0	3630/3386	0.165	0	0.277	0.1020	3579
2	20120308_Porsmilin_PHO_L93_IGN69_0.5	3/8/2012	98	MLWS ->	DRELIO	Nikon D700	77	101	8.46	35	17.1	14	0.042	1	0.099	0.1	0.5	41634	447/0	465/0	0.187	0	0.112	0.0460	11932
3	20150616_Porsmilin_PHO_L93_IGN69_0.5	6/16/2015	87	MLWN ->	DRELIO	Nikon D800	108	72	4.89	35	14.5	9	0.018	0	N.A.	0.029	0.5	45789	415/1	434/1		0.064	0.053	0.0060	11235
4	20170330_Porsmilin_PHO_L93_IGN69_0.5	3/30/2017	109	MLWN ->	DRELIO	Nikon D800	86	31	6.52	35	15.2	17	0.016	0	N.A.	0.0304	0.5	28044	329/1	341/2		-0.053	0.134	0.0300	7380
5	20190122_Porsmilin_PHO_L93_IGN69_0.5	1/22/2019	105	MLWN ->	P4	FC6310R	74	231	2.41	8.8 (24)	18.2	7	0.02	25	0.033	0.1	0.5	50260	684/7	692/7		-0.035	0.038	0.0130	16515
6	20191128_Porsmilin_PHO_L93_IGN69_0.5	11/28/2019	96	MLWN ->	P4 RTK	FC6310R	85	152	2.41	8.8 (24)	20.6	12	0.008	12	0.019	0.1	0.5	43332	617/13	655/51		-0.05	0.043	0.0190	16500

**Online-only Table 4:** PHO Orthophotos and DEMs.

	General information				Data collection											Data processing		Technical validation							
	Name of orthophoto	Date	Tide coeff	Beach coverage	Drone	Camera	Altitude	Number of camera stations	Pixel pitch	Focal length	Ground pixel size	Number of GCPs	RMSE GCPs	Number of ChkPts	RMSE ChkPts	Final grid spacing	Final area	X correction	Y correction	MEx	MEy	MExy	SDExy	Scale	Rotation along vertical axis
							(m)		(μm)	(mm)	(mm)		(m)		(m)	(m)	(m^2^)	(m)	(m)	(m)	(m)	(m)	(m)	(%)	(deg)
1	20091001_Porsmilin_PHO_ORTHO_0.1.tif	10/1/2009	60	MSL ->	DRELIO	Nikon D200	98	66	6.25	35	16.1	50	0.032	2	0.024	0.1	18974			-0.052	0.011	0.069	0.031	100.071	0.015
2	20120308_Porsmilin_PHO_ORTHO_0.1.tif	3/8/2012	98	MLWS ->	DRELIO	Nikon D700	77	101	8.46	35	17.1	14	0.042	1	0.099	0.1	41634	0.881	0.562	0	0	0.093	0.074	100.034	-0.024
3	20150616_Porsmilin_PHO_ORTHO_0.1.tif	6/16/2015	87	MLWN ->	DRELIO	Nikon D800	108	72	4.89	35	14.5	9	0.018	0	N.A.	0.1	45789			0.047	-0.033	0.084	0.06	99.95	-0.011
4	20170330_Porsmilin_PHO_ORTHO_0.1.tif	3/30/2017	109	MLWN ->	DRELIO	Nikon D800	86	31	6.52	35	15.2	17	0.016	0	N.A.	0.1	28044			-0.02	0.031	0.046	0.017	99.975	0
5	20190122_Porsmilin_PHO_ORTHO_0.1.tif	1/22/2019	105	MLWN ->	P4	FC6310R	74	231	2.41	8.8 (24)	18.2	7	0.02	25	0.033	0.1	50260			-0.02	0.044	0.061	0.043	99.977	-0.002
6	20191128_Porsmilin_PHO_ORTHO_0.1.tif	11/28/2019	96	MLWN ->	P4 RTK	FC6310R	85	152	2.41	8.8 (24)	20.6	12	0.008	12	0.019	0.1	43332			-0.007	-0.001	0.034	0.023	99.968	-0.001

**Online-only Table 5:** MES DEMs.

	General information					Data collection				DEM processing					DEM validation					
	Name of DEM file	Date	Tidal coeff.	Coverage	Fusion	Vessel	Sonar	GPS	IMU	Initial grid spacing	Final grid spacing	Final area	Number of filtered DEM cells (all/sand)	Number of filled DEM cells (all/sand)	Vertical correction	ME	SDE	Tilt	Number of DEM cells used for validation	Comments
										(m)	(m)	(m^2^)			(m)	(m)	(m)	(deg)		
1	20080915_Porsmilin_MES_L93_IGN69_0.5	9/15/2008	94	-10 m - > MLWN		Panopée	RESON SeaBat 8160	Septentrio	iXblue Octans	1	0.5	154990	1539/5	5610/419		0.077	0.057	0.002	38032	
2	20081015_Porsmilin_MES_L93_IGN69_0.5	10/15/2008	102	-13 m - > MSL		Panopée	RESON SeaBat 8160	Septentrio	iXblue Octans	1	0.5	249866	2465/3	10473/1297		0.002	0.048	0	65718	
3	20081128_Porsmilin_MES_L93_IGN69_0.5	11/28/2008	75	-12 m- > MSL		Panopée	RESON SeaBat 8160	Septentrio	iXblue Octans	1	0.5	175190	1992/7	9419/1430		0.047	0.05	0.001	43714	
4	20090625_Porsmilin_MES_L93_IGN69_0.5	6/25/2009	97	-7 m - > MSL	x	Panopée	RESON SeaBat 8160	Septentrio	iXblue Octans	1	0.5	236800	1966/1	8284/60		0.066	0.344	N.A.	23572	
5	20090918_Porsmilin_MES_L93_IGN69_0.5	9/18/2009	103	-13 m - > MLWN		Panopée	RESON SeaBat 8160	Septentrio	iXblue Octans	1	0.5	257012	2944/7	5144/76	0.272	0	0.069	0	69366	
6	20100201_Porsmilin_MES_L93_IGN69_0.5	2/1/2010	112	-13 m - > MSL		Panopée	RESON SeaBat 8160	Septentrio	iXblue Octans	1	0.5	419244	5432/8	18836/1356		0.006	0.039	0.002	106166	
7	20100527_Porsmilin_MES_L93_IGN69_0.5	5/27/2010	83	-6 m- > MLWN	x	Albert Lucas	RESON SeaBat 8160	Septentrio	iXblue Octans	1	0.5	61167	808/9	2303/226		0.04	0.037	N.A.	14190	
8	20101104_Porsmilin_MES_L93_IGN69_0.5	11/4/2010	90	-12 m - > MLWN	x	Albert Lucas	RESON SeaBat 8160	Septentrio	iXblue Octans	1	0.5	227910	2993/14	4433/38		-0.056	0.041	0.006	65297	
9	20101122_Porsmilin_MES_L93_IGN69_0.5	11/22/2010	83	-14 m - > MLWN	x	Albert Lucas	RESON SeaBat 8160	Septentrio	iXblue Octans	1	0.5	600302	6837/14	21867/1451	0.095	-0.051	0.042	0.001	127129	
10	20110419_Porsmilin_MES_L93_IGN69_0.5	4/19/2011	109	-14 m - > MLWN	x	Albert Lucas	RESON SeaBat 8160	Septentrio	iXblue Octans	0.5	0.5	450584	5459/27	13638/1347	0.097	-0.058	0.049	0.011	128469	
11	20111124_Porsmilin_MES_L93_IGN69_0.5	11/24/2011	97	-13 m - > CL4		Albert Lucas	RESON SeaBat 8160	Septentrio	iXblue Octans	1	0.5	363232	4486/22	69224/15042		0.007	0.043	0.002	110471	Hole upper shoreface (filled)
12	20121114_Porsmilin_MES_L93_IGN69_0.5	11/14/2012	106	-13 m - > LAT		Albert Lucas	KONGSBERG EM 3002	Ashtec	iXblue Octans	0.5	0.5	561632	11039/11	16652/2603		0.062	0.042	0.002	122160	
13	20131007_Porsmilin_MES_L93_IGN69_0.5	10/7/2013	97	-13 m - > MLWS		Albert Lucas	KONGSBERG EM 3002	Ashtec	iXblue Octans	0.5	0.5	579668	7624/32	171295/10203	-0.085	0	0.049	0.005	119266	Small holes between tracks (filled)
14	20140130_Porsmilin_MES_L93_IGN69_0.5	1/30/2014	102	-13 m - > MLWS	x	Albert Lucas	KONGSBERG EM 3002	Ashtec	iXblue Octans	0.5	0.5	627516	7041/50	56227/1951		0.027	0.052	0.006	127527	
15	20140428_Porsmilin_MES_L93_IGN69_0.5	4/28/2014	95	-14 m - > MLWN		Albert Lucas	KONGSBERG EM 3002	Ashtec	iXblue Octans	0.5	0.5	641974	11350/32	11800/101		0.04	0.053	0.001	130034	
16	20140613_Porsmilin_MES_L93_IGN69_0.5	6/13/2014	94	-14 m - > MLWN	x	Albert Lucas	KONGSBERG EM 3002	Ashtec	iXblue Octans	0.5	0.5	635741	6586/25	28252/7083		0.027	0.077	0.003	128617	Hole lower shoreface (filled)
17	20141124_Porsmilin_MES_L93_IGN69_0.5	11/24/2014	92	-14 m - > MLWS	x	Albert Lucas	KONGSBERG EM 3002	Ashtec	iXblue Octans	0.5	0.5	772857	8660/54	59158/2389	0.148	-0.106	0.084	0.019	122994	
18	20150504_Porsmilin_MES_L93_IGN69_0.5	5/4/2015	85	-14 m - > MLWS	x	Albert Lucas	KONGSBERG EM 3002	Ashtec	iXblue Octans	0.5	0.5	903390	14581/9	25391/2808		0.073	0.102	0.007	130230	
19	20160324_Porsmilin_MES_L93_IGN69_0.5	3/24/2016	89	-12 m - > MLWS		Albert Lucas	KONGSBERG EM 3002	Ashtec	iXblue Octans	0.5	0.5	456271	7176/25	7553/80		0.024	0.045	0.003	130637	
20	20161003_Porsmilin_MES_L93_IGN69_0.5	10/3/2016	87	-12 m - > MLWS		Albert Lucas	KONGSBERG EM 3002	Ashtec	iXblue Octans	0.5	0.5	471595	5633/23	28585/1099		-0.029	0.039	0.001	128989	
21	20161103_Porsmilin_MES_L93_IGN69_0.5	11/3/2016	76	-12 m - > MLWS		Albert Lucas	KONGSBERG EM 3002	Ashtec	iXblue Octans	0.5	0.5	462682	5234/26	30296/270		-0.018	0.042	0.007	129695	
22	20170427_Porsmilin_MES_L93_IGN69_0.5	4/27/2017	109	-12 m - > MLWN		Albert Lucas	KONGSBERG EM 3002	Ashtec	iXblue Octans	0.5	0.5	463982	5922/27	17552/360		-0.049	0.041	0.001	130457	
23	20171204_Porsmilin_MES_L93_IGN69_0.5	12/4/2017	105	-12 m - > MLWN	x	Albert Lucas	KONGSBERG EM 3002	Ashtec	iXblue Octans	0.5	0.5	680988	7676/49	61043/12500		-0.032	0.21	0.01	128068	Hole upper shoreface (filled)
24	20190408_Porsmilin_MES_L93_IGN69_0.5	4/8/2019	88	-12 m - > MLWS	x	Albert Lucas	KONGSBERG EM 3002	Ashtec	iXblue Octans	0.5	0.5	470666	5296/24	21699/795	-0.156	0.078	0.039	0.004	128559	

**Online-only Table 6:** FUS DEMs.

	General information			Source files				DEM validation		
	Name DEM file	Cellsize	Area	Name bathymetric DEM	Date	Name topographic DEM	Date	ME	SDE	Number of DEM cells used for validation
		(m)	(m2)					(m)	(m)	
1	20090625_Porsmilin_FUS_L93_IGN69_0.5	0.5	259686	20090625_Porsmilin_MES_L93_IGN69_0.5	25/06/2009	20090625_Porsmilin_TLS_L93_IGN69_0.5	25/06/2009	-0.019	0.04	39824
2	20100527_Porsmilin_FUS_L93_IGN69_0.5	0.5	88450	20100527_Porsmilin_MES_L93_IGN69_0.5	27/05/2010	20100603_Porsmilin_TLS_L93_IGN69_0.5	03/06/2010	-0.065	0.102	10479
3	20101104_Porsmilin_FUS_L93_IGN69_0.5	0.5	252956	20101104_Porsmilin_MES_L93_IGN69_0.5	04/11/2010	20101104_Porsmilin_TLS_L93_IGN69_0.5	04/11/2010	0.054	0.04	16442
4	20101122_Porsmilin_FUS_L93_IGN69_0.5	0.5	624142	20101122_Porsmilin_MES_L93_IGN69_0.5	22/11/2010	20101118_Porsmilin_TLS_L93_IGN69_0.5	18/11/2010	0	0.032	508
5	20110419_Porsmilin_FUS_L93_IGN69_0.5	0.5	474588	20110419_Porsmilin_MES_L93_IGN69_0.5	19/04/2011	20110420_Porsmilin_TLS_L93_IGN69_0.5	20/04/2011	0.008	0.06	20807
6	20140130_Porsmilin_FUS_L93_IGN69_0.5	0.5	663358	20140130_Porsmilin_MES_L93_IGN69_0.5	30/01/2014	20140130_Porsmilin_TLS_L93_IGN69_0.5	30/01/2014	-0.043	0.064	8344
7	20140613_Porsmilin_FUS_L93_IGN69_0.5	0.5	663928	20140613_Porsmilin_MES_L93_IGN69_0.5	13/06/2014	20140612_Porsmilin_TLS_L93_IGN69_0.5	12/06/2014	-0.084	0.101	16034
8	20141124_Porsmilin_FUS_L93_IGN69_0.5	0.5	805699	20141124_Porsmilin_MES_L93_IGN69_0.5	24/11/2014	20141124_Porsmilin_TLS_L93_IGN69_0.5	24/11/2014	-0.038	0.044	8599
9	20150504_Porsmilin_FUS_L93_IGN69_0.5	0.5	939632	20150504_Porsmilin_MES_L93_IGN69_0.5	04/05/2015	20150504_Porsmilin_TLS_L93_IGN69_0.5	04/05/2015	−0.003	0.05	6253
10	20171204_Porsmilin_FUS_L93_IGN69_0.5	0.5	715084	20171204_Porsmilin_MES_L93_IGN69_0.5	04/12/2017	20171205_Porsmilin_TLS_L93_IGN69_0.5	05/12/2017	0.122	0.08	21066
11	20190408_Porsmilin_FUS_L93_IGN69_0.5	0.5	504415	20190408_Porsmilin_MES_L93_IGN69_0.5	08/04/2019	20190409_Porsmilin_TLS_L93_IGN69_0.5	09/04/2019	−0.007	0.083	3232

**Online-only Table 7:** FUS profiles.

	General information		Source files				Technical validation		
	Name fusion profile	Length	Name bathymetric profile	Date	Name topographic profile	Date	ME	SDE	Profile length where data overlap
		(m)					(m)	(m)	(m)
1	20081015_Porsmilin_FUS_L93_IGN69_0.5_P	664	20081015_Porsmilin_MES_Dist_IGN69_0.5_D	15/10/2008	20081013_Porsmilin_GPS_Dist_IGN69_0.5_P	13/10/2008	−0.005	0.028	114
2	20081128_Porsmilin_FUS_L93_IGN69_0.5_P	633	20081128_Porsmilin_MES_Dist_IGN69_0.5_D	28/11/2008	20081114_Porsmilin_GPS_Dist_IGN69_0.5_P	14/11/2008	−0.005	0.078	153
3	20100201_Porsmilin_FUS_L93_IGN69_0.5_P	840	20100201_Porsmilin_MES_Dist_IGN69_0.5_D	01/02/2010	20100129_Porsmilin_GPS_Dist_IGN69_0.5_P	29/01/2010	−0.034	0.04	153
